# Association between the triglyceride-glucose index and the presence and severity of hemorrhoidal disease: a cross-sectional study based on retrospectively collected clinical data

**DOI:** 10.3389/fendo.2026.1935994

**Published:** 2026-08-31

**Authors:** Tingting Li, Li Jiang, Hanwen Yang, Jianan Li

**Affiliations:** 1 Department of Proctology, China-Japan Friendship Hospital, Beijing, China; 2 Diabetes Department of Integrated Chinese and Western Medicine, China-Japan Friendship Hospital, Beijing, China

**Keywords:** cross-sectional study, hemorrhoidal disease, insulin resistance, metabolic dysfunction, restricted cubic spline, triglyceride-glucose index

## Abstract

**Objective:**

To investigate the association between the triglyceride-glucose (TyG) index and the prevalence and severity of hemorrhoidal disease in adults undergoing anorectal evaluation.

**Methods:**

This cross-sectional study analyzed retrospectively collected data from 528 adults undergoing anorectal evaluation and fasting biochemical testing. The TyG index was calculated as ln[fasting triglycerides (mg/dL) × fasting plasma glucose (mg/dL)/2]. Multivariable logistic regression, restricted cubic spline analysis, subgroup, sensitivity, severity, and exploratory mediation analyses were performed to assess the association between TyG and hemorrhoidal disease.

**Results:**

Among the 528 participants, 196 (37.1%) had hemorrhoidal disease. Its prevalence increased progressively across TyG quartiles, from 23.5% in Q1 to 53.8% in Q4 (P for trend<0.001). After adjustment for demographic, metabolic, lifestyle, medication-related, and bowel-related factors, each 1-unit increase in the TyG index was associated with higher odds of hemorrhoidal disease (OR = 1.68, 95% CI: 1.24–2.28; P = 0.001). Compared with Q1, participants in Q4 had significantly greater odds of hemorrhoidal disease (OR = 2.43, 95% CI: 1.34–4.41; P for trend=0.002). Restricted cubic spline analysis showed a significant overall association (P<0.001) without evidence of nonlinearity (P = 0.184). The findings remained consistent across subgroup and sensitivity analyses. A higher TyG index was also associated with advanced hemorrhoidal disease (OR per 1-unit increase=1.62, 95% CI: 1.01–2.61).

**Conclusion:**

A higher TyG index was independently associated with hemorrhoidal disease severity. BMI partially mediated this association (~27%), suggesting a potential metabolic pathway that requires validation in longitudinal studies.

## 1 Introduction

Hemorrhoidal disease is one of the most common anorectal disorders and represents a substantial clinical and public health burden (1). It is characterized by symptomatic enlargement and distal displacement of the normal anal cushions and may present with rectal bleeding, prolapse, anal discomfort, pruritus, or pain. Although hemorrhoidal disease is generally benign, recurrent symptoms can markedly impair quality of life and increase healthcare utilization (1). Its development is considered multifactorial. Conventional risk factors include chronic constipation, prolonged straining during defecation, low dietary fiber intake, sedentary behavior, obesity, pregnancy, aging, and occupations involving prolonged sitting or standing (2). However, these traditional explanations may not fully account for the considerable interindividual variation in disease susceptibility and severity.

Growing evidence suggests that systemic metabolic abnormalities may also contribute to the development of anorectal vascular disorders. Insulin resistance, dyslipidemia, obesity, and chronic low-grade inflammation are associated with endothelial dysfunction, impaired microcirculation, oxidative stress, and extracellular matrix remodeling (3). These pathophysiological changes may alter venous tone and blood flow in the hemorrhoidal plexus, increase vascular congestion, and weaken the supporting connective tissues of the anal cushions. In addition, metabolic dysfunction may coexist with behavioral factors such as physical inactivity, constipation, and unhealthy dietary patterns, thereby creating a biological and lifestyle environment conducive to hemorrhoidal disease (4). Nevertheless, the potential association between metabolic impairment and hemorrhoidal disease has received limited attention.

The triglyceride-glucose index is a simple surrogate marker of insulin resistance calculated from fasting triglyceride and fasting plasma glucose concentrations (4).

Compared with direct measurements of insulin resistance, such as the hyperinsulinemic-euglycemic clamp, the TyG index is inexpensive, easily calculated, and widely available in routine clinical practice (5). Previous studies have demonstrated that a higher TyG index is associated with obesity, metabolic syndrome, type 2 diabetes, nonalcoholic fatty liver disease, cardiovascular disease, endothelial dysfunction, and adverse microvascular outcomes (6). These findings indicate that the TyG index may capture clinically relevant disturbances in glucose and lipid metabolism that extend beyond conventional metabolic diagnoses.

Despite the increasing application of the TyG index in metabolic and cardiovascular research, its relationship with hemorrhoidal disease remains unclear. Existing studies of hemorrhoidal disease have primarily focused on bowel habits, dietary patterns, pregnancy, aging, and local anatomical or mechanical factors. Few investigations have examined whether systemic insulin resistance and combined glucose-lipid abnormalities are associated with the presence or clinical severity of hemorrhoidal disease. Moreover, the potential dose-response pattern between the TyG index and hemorrhoidal disease has not been adequately characterized, and it remains uncertain whether the association is linear or whether a threshold effect exists (7).

Accordingly, the present retrospective cross-sectional study aimed to investigate the association between the TyG index and hemorrhoidal disease. We further examined the dose-response relationship using restricted cubic spline analysis and evaluated whether the association differed across clinically relevant subgroups defined by age, sex, body mass index, diabetes status, and constipation. Furthermore, while constipation and obesity are established risk factors for hemorrhoidal disease, it remains unclear whether they act as confounders or as intermediate mediators in the pathway from metabolic dysfunction to hemorrhoidal disease. Therefore, we also aimed to explore whether the TyG-hemorrhoid association, if present, was mediated by increased BMI or constipation. We hypothesized that a higher TyG index would be independently associated with greater odds of hemorrhoidal disease and possibly with more advanced disease severity. These findings provide preliminary epidemiological evidence of an association between systemic metabolic dysfunction and hemorrhoidal disease among clinically evaluated adults, while further population-based studies are required to determine whether this relationship extends to broader populations.

## 2 Materials and methods

### 2.1 Study design and participants

This cross-sectional study was based on retrospectively collected electronic health record data from the Department of Proctology, China-Japan Friendship Hospital, Beijing, China. Consecutive adults who underwent clinically indicated anorectal evaluation and fasting biochemical testing between August 2024 and February 2026 were screened for eligibility. The study was designed to investigate the association between the TyG index and the presence of hemorrhoidal disease.

Participants were eligible if they met all of the following criteria: (1) age ≥18 years; (2) completion of a standardized anorectal examination, including visual inspection, digital rectal examination, and anoscopy or proctoscopy; and (3) availability of fasting plasma glucose and fasting triglyceride measurements obtained during the same clinical evaluation period.

The exclusion criteria were: (1) a history of colorectal or major anorectal surgery; (2) inflammatory bowel disease, colorectal malignancy, anal fistula, perianal abscess, anal tuberculosis, or other conditions that could interfere with the diagnosis of hemorrhoidal disease; (3) severe hepatic dysfunction, end-stage renal disease, acute infection, or other serious systemic illness; (4) pregnancy or lactation; (5) acute thrombosed external hemorrhoids requiring emergency treatment; (6) missing core exposure data (fasting glucose or triglyceride concentrations required for TyG calculation) or missing outcome assessment of hemorrhoidal disease; and (7) insufficient clinical information, defined as missingness exceeding 30% among prespecified covariates, which was considered inadequate for reliable multivariable adjustment. Participants were classified into the hemorrhoidal disease group and the non-hemorrhoidal disease group according to standardized anorectal assessment. Because recruitment was performed at a specialized proctology center, the study population consisted of adults undergoing clinical anorectal evaluation rather than individuals from a community screening cohort.

### 2.2 Diagnostic assessment of hemorrhoidal disease

The presence of hemorrhoidal disease was determined by experienced colorectal specialists based on clinical symptoms and standardized anorectal examination. Diagnostic assessment included perianal inspection, digital rectal examination, and anoscopy or proctoscopy where clinically appropriate.

Hemorrhoidal disease was diagnosed when enlarged or prolapsed hemorrhoidal cushions were identified together with compatible clinical manifestations, including painless rectal bleeding, prolapse, anal discomfort, pruritus, mucus discharge, or pain. Participants without clinical or endoscopic evidence of hemorrhoidal disease were classified as controls.

Internal hemorrhoids were graded according to the Goligher classification:

- Grade I: hemorrhoidal cushions without prolapse;
- Grade II: prolapse during defecation with spontaneous reduction;
- Grade III: prolapse requiring manual reduction;
- Grade IV: permanently prolapsed and irreducible hemorrhoids.

For the primary analysis, hemorrhoidal disease was defined as the presence of clinically diagnosed hemorrhoidal disease, including internal, external, and mixed hemorrhoids. For the secondary severity analysis, only participants with classifiable internal hemorrhoidal components were included. Disease severity was categorized according to the Goligher classification, with grade I–II considered early-stage disease and grade III–IV considered advanced disease. External hemorrhoids without a gradable internal component were excluded from the severity analysis because the Goligher system does not adequately classify isolated external disease. External and mixed hemorrhoids were recorded separately. When the prolapsing internal component of mixed hemorrhoids could be graded, the Goligher grade was assigned according to the internal component.

All diagnoses were independently reviewed by two colorectal specialists. Disagreements were resolved through consensus review. Examiners were unaware of the calculated TyG index at the time of outcome classification whenever possible.

### 2.3 Laboratory measurements and TyG index calculation

Venous blood samples were collected after an overnight fast of at least 8 hours. Fasting plasma glucose and triglyceride concentrations were measured using standard enzymatic methods with an automated biochemical analyzer (cobas 8000 c702, Roche Diagnostics, Mannheim, Germany) in the hospital’s central laboratory. Total cholesterol, high-density lipoprotein cholesterol, low-density lipoprotein cholesterol, uric acid, alanine aminotransferase, aspartate aminotransferase, serum creatinine, and other available biochemical variables were also recorded.

For participants with repeated measurements, laboratory results obtained on the same day as the anorectal assessment were preferentially selected. When same-day measurements were unavailable, the closest fasting measurement obtained within 14 days before or after the anorectal examination was used, provided that no major treatment change or acute illness occurred during this interval.

The TyG index was calculated using the following formula:


$$\text{TyG index}=\ln\left[\frac{\text{fasting triglycerides}\left(\text{mg/dL}\right)\times \text{fasting plasma glucose}\left(\text{mg/dL}\right)}{2}\right]$$

When laboratory values were originally reported in mmol/L, triglycerides were converted to mg/dL by multiplying by 88.57, and fasting plasma glucose was converted to mg/dL by multiplying by 18.0.

The TyG index was analyzed both as a continuous variable and as a categorical variable according to quartiles. The lowest quartile was used as the reference category in regression analyses.

### 2.4 Covariates

Demographic, anthropometric, clinical, lifestyle, medication-related, and bowel-habit variables were extracted from electronic medical records. The final adjustment set for the primary analysis (Model 3) included: age (continuous, years); sex (male/female); body mass index (continuous, kg/m²); smoking status (categorical: current/former/never); alcohol consumption (binary: yes/no); hypertension (binary: yes/no); diabetes mellitus (binary: yes/no); use of glucose-lowering medications (binary: yes/no); use of lipid-lowering medications (binary: yes/no); constipation (binary: yes/no, based on documented clinical history or standardized assessment); sedentary behaviour (binary: yes/no, defined as ≥6 h/day of sitting); physical activity (binary: yes/no, defined as ≥150 min/week of moderate-intensity activity); dietary fibre intake (binary: adequate/inadequate, based on dietary recall); chronic diarrhoea (binary: yes/no); straining during defecation (binary: yes/no); and prolonged toilet sitting (binary: yes/no, defined as ≥15 min per episode). The complete list of candidate covariates considered *a priori*, together with definitions and selection criteria, is provided in **Supplementary Table 1**.

Body mass index was calculated as weight in kilograms divided by height in meters squared. Hypertension and diabetes mellitus were identified from documented physician diagnoses, medication records, or available clinical measurements according to the definitions used in the hospital database.

Constipation was defined according to documented clinical history or standardized bowel habit assessment, including infrequent defecation, hard stools, excessive straining, a sensation of incomplete evacuation, or the use of laxatives. Prolonged toilet sitting was defined as an average defecation duration of ≥15 minutes, where this information was available.

Because fasting glucose and triglyceride concentrations are direct components of the TyG index, these two variables were not simultaneously entered with TyG in any regression model. Additional lipid-related biomarkers (total cholesterol, HDL-C, and LDL-C) were evaluated only in the extended model after assessment of multicollinearity.

Of note, BMI and constipation were treated as candidate mediators rather than confounders in the mediation models. Therefore, they were not simultaneously adjusted as covariates when serving as the mediator in the respective models, to avoid overadjustment for intermediate variables.

### 2.5 Statistical analysis

All statistical analyses were performed using R software (version 4.4.1) and IBM SPSS Statistics (version 26.0). A two-sided *P* < 0.05 was considered statistically significant. Continuous variables were compared using the independent-samples t-test or Mann–Whitney *U* test, and categorical variables using the χ² test or Fisher’s exact test, as appropriate.

The TyG index was analysed as a continuous variable (per 1-unit and per 1-standard-deviation [SD] increase) and categorised into quartiles (Q1–Q4), with Q1 as the reference. Binary logistic regression was used to estimate odds ratios (ORs) and 95% confidence intervals (CIs) for the presence of hemorrhoidal disease. Three sequential models were fitted: Model 1, unadjusted; Model 2, adjusted for age and sex; and Model 3 (primary model). Model 4 (extended model) further adjusted for total cholesterol, high-density lipoprotein cholesterol, low-density lipoprotein cholesterol, and uric acid to test robustness. Because fasting glucose and triglycerides are direct components of the TyG index, they were not simultaneously entered with TyG in any regression model. Variance inflation factors (VIFs) were calculated for all models; the maximum VIF was 2.41 in Model 4, below the prespecified threshold of 5, indicating no problematic multicollinearity.

Missing data in covariates (all ≤4.0%) were handled using multiple imputation by chained equations (MICE) under the missing-at-random assumption, generating 20 imputed datasets. The primary outcome and TyG components were not imputed. Complete-case analysis was performed as a sensitivity analysis.

Restricted cubic spline regression with four knots (at the 5th, 35th, 65th, and 95th percentiles of the TyG distribution) was used to examine the dose-response shape, with the median TyG (8.62) as the reference; overall association and nonlinearity were tested by Wald tests.

Prespecified subgroup analyses were performed according to sex, age (<45 vs ≥45 years), BMI (<24 vs ≥24 kg/m²), diabetes status, and constipation status. Multiplicative interaction terms between the continuous TyG index and each subgroup variable were added to Model 3 to test effect modification.

For the severity analysis, only participants with classifiable internal hemorrhoidal components (Goligher grade I–IV) were included; advanced disease (grade III–IV) was compared with early-stage disease (grade I–II) using logistic regression with the same Model 3 covariates.

Exploratory mediation analyses using the ‘mediation’ package (version 4.5.0) examined BMI (continuous) and constipation (binary) as candidate mediators, estimating the average causal mediation effect (ACME) and average direct effect (ADE) with 1,000 quasi-Bayesian simulations. All mediation models were adjusted for Model 3 covariates except the mediator itself. Given the cross-sectional design, these analyses are hypothesis-generating and do not imply causal mediation.

Sensitivity analyses were conducted by: (1) excluding lipid-lowering medication users; (2) excluding participants with diabetes or glucose-lowering medication users; (3) excluding extreme TyG values (<1st or >99th percentile); and (4) complete-case analysis.

## 3 Result

### 3.1 Participant selection

A total of 684 consecutive adults who underwent anorectal evaluation and fasting biochemical testing between August 2024 and February 2026 were initially screened. Of these, 156 participants were excluded: 42 had incomplete fasting triglyceride or glucose measurements required for TyG calculation, 31 had a history of colorectal or major anorectal surgery, 28 had concomitant anorectal diseases that could interfere with hemorrhoidal assessment, 17 had inflammatory bowel disease or colorectal malignancy, 14 had severe hepatic or renal dysfunction, 8 were pregnant or lactating, and 16 had insufficient clinical information for reliable adjustment. Specifically, these 16 participants were excluded because missing information exceeded the prespecified threshold of 30% among key covariates or involved essential clinical variables required for multivariable modeling.(**Supplementary Table 2**).

**Figure 1:**
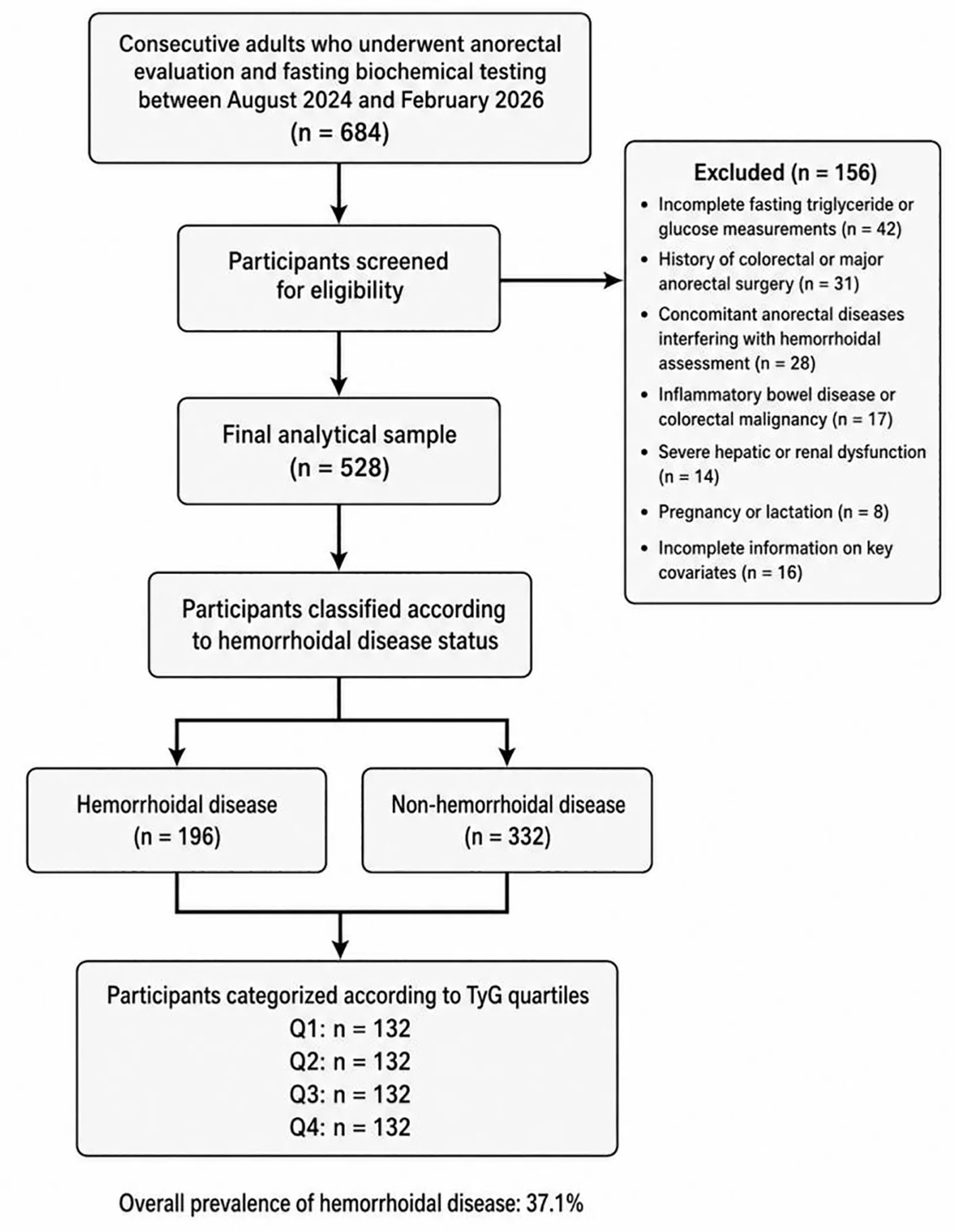
Flowchart of participant selection.

The final analytical sample comprised 528 participants, including 196 participants with hemorrhoidal disease and 332 participants without hemorrhoidal disease. The detection rate of hemorrhoidal disease among adults undergoing anorectal evaluation was 37.1%. This proportion represents the prevalence observed in this clinically evaluated cohort and should not be interpreted as a population-based prevalence estimate. Participants were divided into four equal groups according to the quartile distribution of the TyG index, with 132 participants in each quartile. The participant selection process is shown in **Figure 1**.

No prespecified covariate had missingness exceeding 20%. Therefore, all eligible missing covariate values were handled using MICE in the primary analysis.

### 3.2 Baseline characteristics by TyG quartiles

The median TyG index in the overall population was 8.62, with an interquartile range of 8.22–9.03. Participants were categorized into the following quartiles: Q1, TyG<8.22; Q2, 8.22–8.61; Q3, 8.62–9.02; and Q4, ≥9.03.

Baseline characteristics according to TyG quartiles are presented in **Table 1**. Participants in the highest TyG quartile were older and had higher body mass index, systolic blood pressure, fasting plasma glucose, triglyceride, total cholesterol, low-density lipoprotein cholesterol, and uric acid levels than those in the lowest quartile. The prevalence of hypertension, diabetes mellitus, constipation, sedentary behavior, and hemorrhoidal disease also increased across TyG quartiles.

**Table 1:** Baseline characteristics of participants according to TyG index quartiles.

Variable	Q1, <8.22 (n=132)	Q2, 8.22–8.61 (n=132)	Q3, 8.62–9.02 (n=132)	Q4, ≥9.03 (n=132)	P value
TyG index	7.91 ± 0.22	8.43 ± 0.11	8.81 ± 0.12	9.38 ± 0.31	<0.001
Age, years	42.7 ± 11.8	45.1 ± 12.0	47.3 ± 11.4	50.2 ± 10.9	<0.001
Male, n (%)	72 (54.5)	75 (56.8)	77 (58.3)	80 (60.6)	0.753
BMI, kg/m²	22.3 ± 2.6	23.7 ± 2.8	25.1 ± 3.0	27.0 ± 3.2	<0.001
Systolic blood pressure, mmHg	119.6 ± 13.4	123.1 ± 14.1	127.3 ± 15.0	132.8 ± 15.6	<0.001
Fasting plasma glucose, mmol/L	4.82 ± 0.46	5.06 ± 0.58	5.39 ± 0.71	6.21 ± 1.28	<0.001
Triglycerides, mmol/L	0.82 (0.69–0.96)	1.14 (1.02–1.27)	1.53 (1.36–1.72)	2.31 (1.94–2.89)	<0.001
Total cholesterol, mmol/L	4.32 ± 0.71	4.55 ± 0.78	4.79 ± 0.83	5.08 ± 0.91	<0.001
HDL-C, mmol/L	1.42 ± 0.31	1.34 ± 0.29	1.25 ± 0.27	1.13 ± 0.25	<0.001
LDL-C, mmol/L	2.48 ± 0.59	2.66 ± 0.64	2.83 ± 0.68	3.02 ± 0.73	<0.001
Uric acid, μmol/L	303.7 ± 68.2	326.8 ± 73.5	347.1 ± 77.4	376.5 ± 82.1	<0.001
Current smoking, n (%)	25 (18.9)	29 (22.0)	34 (25.8)	39 (29.5)	0.187
Alcohol consumption, n (%)	21 (15.9)	25 (18.9)	29 (22.0)	35 (26.5)	0.161
Hypertension, n (%)	17 (12.9)	25 (18.9)	36 (27.3)	50 (37.9)	<0.001
Diabetes mellitus, n (%)	4 (3.0)	8 (6.1)	15 (11.4)	30 (22.7)	<0.001
Constipation, n (%)	29 (22.0)	36 (27.3)	44 (33.3)	53 (40.2)	0.009
Sedentary behavior, n (%)	36 (27.3)	43 (32.6)	51 (38.6)	61 (46.2)	0.01
Chronic diarrhea, n (%)	8 (6.1)	9 (6.8)	10 (7.6)	12 (9.1)	0.79
Hemorrhoidal disease, n (%)	31 (23.5)	41 (31.1)	53 (40.2)	71 (53.8)	<0.001

Data are presented as mean ± standard deviation, median (interquartile range), or number (percentage), as appropriate. BMI, body mass index; HDL-C, high-density lipoprotein cholesterol; LDL-C, low-density lipoprotein cholesterol; TyG, triglyceride-glucose.

The prevalence of hemorrhoidal disease was 23.5% in Q1, 31.1% in Q2, 40.2% in Q3, and 53.8% in Q4, showing a significant increasing trend across quartiles (P for trend<0.001). No statistically significant differences were observed in sex distribution or chronic diarrhea across the four groups.

### 3.3 Association between TyG index and hemorrhoidal disease

The results of multivariable logistic regression analyses are shown in **Table 2**. When analysed as a continuous variable, each 1-unit increase in the TyG index was associated with higher odds of hemorrhoidal disease in the unadjusted model (OR = 2.14, 95% CI: 1.66–2.76; *P* < 0.001). After adjustment for age and sex, the OR was 1.95 (95% CI: 1.49–2.56; *P* < 0.001). In the primary adjusted model (Model 3), each 1-unit increase in TyG was associated with an OR of 1.68 (95% CI: 1.24–2.28; *P* = 0.001). When standardised, each 1-SD increase in TyG was associated with a 34% increase in the odds of hemorrhoidal disease (OR = 1.34, 95% CI: 1.14–1.58; *P* < 0.001). Compared with Q1, the fully adjusted ORs were 1.29 (95% CI: 0.73–2.28) for Q2, 1.71 (95% CI: 0.97–3.02) for Q3, and 2.43 (95% CI: 1.34–4.41) for Q4, with a significant dose-response trend (*P* for trend = 0.002). Results from the extended Model 4 were similar (OR per 1-unit increase = 1.61, 95% CI: 1.17–2.21).

**Table 2:** Multivariable logistic regression analyses of the association between TyG index and hemorrhoidal disease.

TyG exposure	Cases/total	Model 1 OR (95% CI)	Model 2 OR (95% CI)	Model 3 OR (95% CI) primary model	Model 4 OR (95% CI) extended model
Per 1-unit increase	196/528	2.14 (1.66–2.76)	1.95 (1.49–2.56)	1.68 (1.24–2.28)	1.61 (1.17–2.21)
Per 1-SD increase	196/528	1.51 (1.31–1.75)	1.45 (1.24–1.69)	1.34 (1.14–1.58)	1.31 (1.11–1.55)
Q1	31/132	Reference	Reference	Reference	Reference
Q2	41/132	1.47 (0.86–2.51)	1.39 (0.81–2.40)	1.29 (0.73–2.28)	1.25 (0.70–2.23)
Q3	53/132	2.19 (1.31–3.65)	1.98 (1.17–3.35)	1.71 (0.97–3.02)	1.65 (0.92–2.95)
Q4	71/132	3.79 (2.28–6.29)	3.21 (1.89–5.46)	2.43 (1.34–4.41)	2.29 (1.24–4.23)
P for trend	—	<0.001	<0.001	0.002	0.004

Model 1 was unadjusted. Model 2 was adjusted for age and sex. Model 3 (primary model) and all subsequent adjusted analyses were adjusted for covariates listed in Methods 2.4 (age, sex, BMI, smoking, alcohol, hypertension, diabetes, medication use, constipation, sedentary behaviour, physical activity, dietary fibre, chronic diarrhoea, straining, and prolonged sitting). Model 4 was additionally adjusted for total cholesterol, HDL-C, LDL-C, and uric acid. TyG, triglyceride-glucose index; HDL-C, high-density lipoprotein cholesterol; LDL-C, low-density lipoprotein cholesterol.

### 3.4 Restricted cubic spline analysis

Restricted cubic spline analysis demonstrated a positive association between the continuous TyG index and the odds of hemorrhoidal disease after adjustment for potential confounders. The overall association was statistically significant (P for overall association<0.001), whereas there was no strong evidence of nonlinearity (P for nonlinearity=0.184).

**Figure 2:**
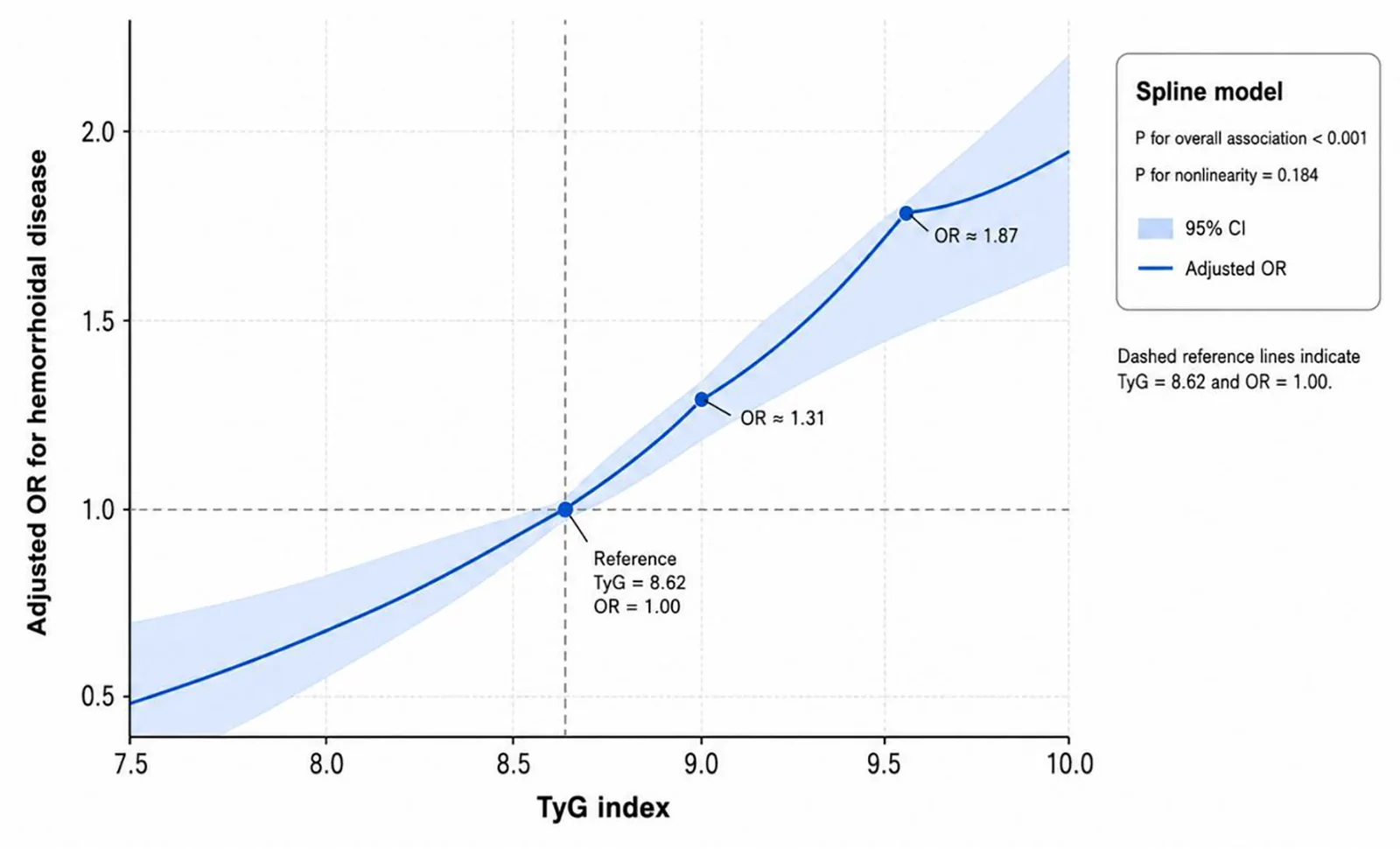
Restricted cubic spline analysis of the association between the TyG index and hemorrhoidal disease.

The estimated odds of hemorrhoidal disease increased gradually as the TyG index increased, particularly above the median value of 8.62. Compared with the reference value of 8.62, the adjusted OR was approximately 1.31 at a TyG index of 9.0 and 1.87 at a TyG index of 9.5. The confidence intervals widened at the lower and upper extremes because of the smaller number of observations in these ranges (**Figure 2**)

These findings suggest that the association between the TyG index and hemorrhoidal disease was approximately linear rather than characterized by a clear threshold effect.

The solid line represents the adjusted odds ratio, and the shaded area represents the 95% confidence interval. The median TyG index of 8.62 was used as the reference. The spline model was adjusted for the covariates included in Model 3 of the primary analysis.

### 3.5 Subgroup and interaction analyses

**Figure 3:**
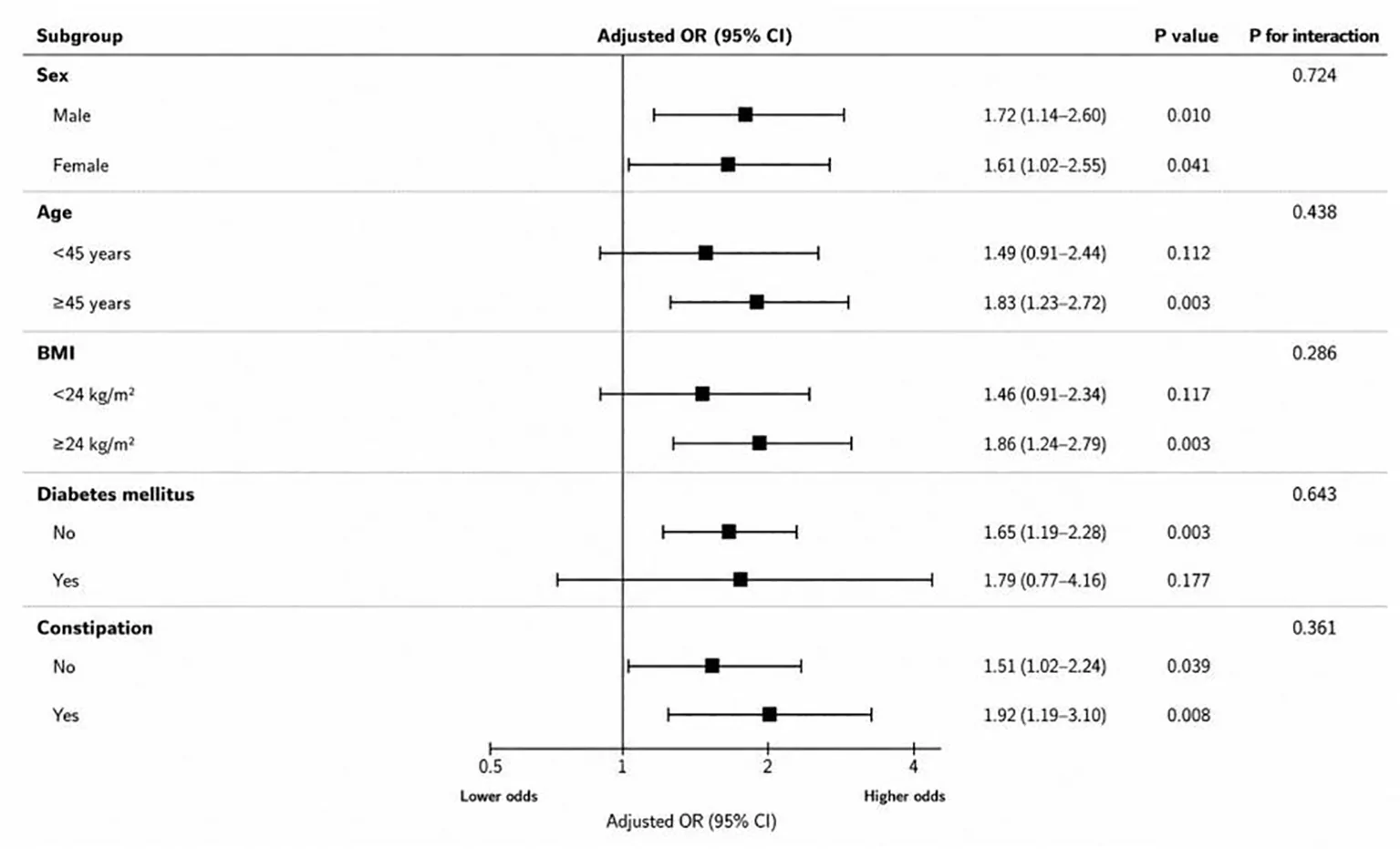
Forest plot of subgroup analyses for the association between the TyG index and hemorrhoidal disease.

Subgroup analyses generally showed a positive association between the TyG index and hemorrhoidal disease across clinically relevant groups (**Figure 3**). Detailed subgroup estimates are provided in **Supplementary Table 3**.

The association appeared stronger among participants aged ≥45 years, those with BMI ≥24 kg/m², and those with constipation. However, no statistically significant interactions were detected for sex, age, BMI, diabetes status, or constipation status, with all P values for interaction exceeding 0.05.

Among participants aged ≥45 years, each 1-unit increase in TyG was associated with an adjusted OR of 1.83 (95% CI: 1.23–2.72), compared with 1.49 (95% CI: 0.91–2.44) among participants aged<45 years (P for interaction=0.438).

Among participants with constipation, the adjusted OR was 1.92 (95% CI: 1.19–3.10), whereas the corresponding OR among those without constipation was 1.51 (95% CI: 1.02–2.24; P for interaction=0.361). These differences should therefore be interpreted as descriptive rather than evidence of statistically confirmed effect modification.

### 3.6 Sensitivity analyses

The association between a higher TyG index and hemorrhoidal disease remained generally consistent across sensitivity analyses (**Table 3**).

**Table 3:** Sensitivity analyses of the association between TyG index and hemorrhoidal disease.

Sensitivity analysis	Participants, n	OR per 1-unit increase (95% CI)	P value	Q4 vs. Q1 OR (95% CI)
Primary analysis	528	1.68 (1.24–2.28)	0.001	2.43 (1.34–4.41)
Excluding lipid-lowering medication users	464	1.72 (1.23–2.41)	0.002	2.51 (1.32–4.78)
Excluding diabetes and glucose-lowering medication users	467	1.59 (1.13–2.24)	0.008	2.17 (1.15–4.11)
Excluding extreme TyG values	518	1.66 (1.21–2.27)	0.002	2.38 (1.30–4.36)
Complete-case analysis	501	1.64 (1.19–2.26)	0.003	2.31 (1.25–4.28)

All models were adjusted for the covariates included in Model 3 of the primary analysis.

After excluding 64 participants receiving lipid-lowering medications, each 1-unit increase in the TyG index was associated with an adjusted OR of 1.72 (95% CI: 1.23–2.41; P = 0.002). After excluding participants with diabetes mellitus or those receiving glucose-lowering medications, the corresponding OR was 1.59 (95% CI: 1.13–2.24; P = 0.008).

Exclusion of participants with TyG values below the 1st percentile or above the 99th percentile yielded an OR of 1.66 (95% CI: 1.21–2.27; P = 0.002). Complete-case analysis also produced a comparable estimate (OR = 1.64, 95% CI: 1.19–2.26; P = 0.003). The primary analysis was conducted in the MICE-imputed dataset (n=528), whereas the complete-case sensitivity analysis included 501 participants with no missing values in any model variables.

The highest TyG quartile remained significantly associated with hemorrhoidal disease in all sensitivity models, with adjusted ORs ranging from 2.17 to 2.51 compared with the lowest quartile. These findings indicated that the primary results were not substantially influenced by medication use, diabetes status, extreme TyG values, or the method used to handle missing data.

### 3.7 Association with disease severity

Among the 196 participants with hemorrhoidal disease, severity analysis was restricted to participants with classifiable internal hemorrhoidal components. A total of 126 participants had grade I–II disease and 70 had grade III–IV disease. The proportion of advanced hemorrhoidal disease increased across TyG quartiles, from 22.6% among affected participants in Q1 to 45.1% among those in Q4 (P for trend=0.018).

In the unadjusted analysis, each 1-unit increase in the TyG index was associated with higher odds of advanced hemorrhoidal disease (OR = 1.78, 95% CI: 1.13–2.80; P = 0.013). After adjustment using the same covariate framework as Model 3 of the primary analysis, the association remained statistically significant (OR = 1.62, 95% CI: 1.01–2.61; P = 0.046).

Compared with patients in the lowest TyG quartile, those in the highest quartile had higher odds of grade III–IV hemorrhoidal disease, although the confidence interval was relatively wide because of the limited number of severity events (adjusted OR = 2.36, 95% CI: 1.01–5.52; P = 0.048) (**Table 4**).

**Table 4:** Association between TyG index and advanced hemorrhoidal disease among affected participants.

TyG exposure	Advanced/total	Unadjusted OR (95% CI)	Adjusted OR (95% CI)
Per 1-unit increase	70/196	1.78 (1.13–2.80)	1.62 (1.01–2.61)
Per 1-SD increase	70/196	1.37 (1.05–1.80)	1.29 (1.00–1.68)
Q1	7/31	Reference	Reference
Q2	11/41	1.26 (0.42–3.75)	1.18 (0.38–3.64)
Q3	20/53	2.08 (0.75–5.79)	1.84 (0.63–5.35)
Q4	32/71	2.81 (1.08–7.34)	2.36 (1.01–5.52)
P for trend	—	0.014	0.031

Advanced hemorrhoidal disease was defined as Goligher grade III-IV, and early-stage disease was defined as grade I-II. The adjusted model used the same covariate framework as Model 3 of the primary analysis and included age, sex, body mass index, smoking status, alcohol consumption, hypertension, diabetes mellitus, glucose-lowering medication use, lipid-lowering medication use, constipation, sedentary behavior, physical activity, dietary fiber intake, chronic diarrhea, straining during defecation, and prolonged toilet sitting.

### 3.8 Mediation analysis

**Figure 4:**
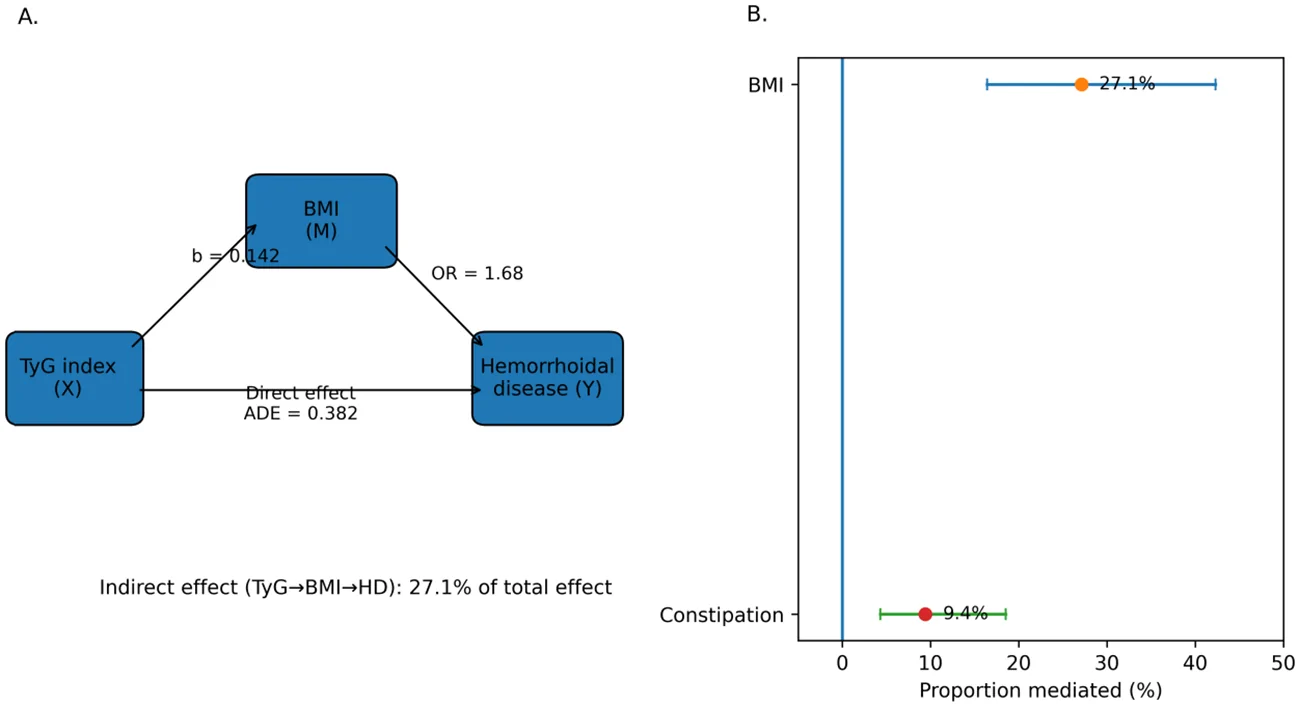
Exploratory mediation analysis of BMI and constipation in the association between TyG index and hemorrhoidal disease. **(A)** Exploratory path diagram illustrating the potential BMI-related pathway linking TyG index and hemorrhoidal disease. Values are presented as regression coefficients and odds ratios. The estimated indirect association through BMI accounted for 27.1% of the total association, while a substantial direct association remained. **(B)** Estimated proportion of association explained by BMI and constipation with 95% confidence intervals. All models were adjusted for age, sex, smoking, alcohol consumption, hypertension, diabetes mellitus, medication use, sedentary behavior, physical activity, and dietary fiber intake. Because of the cross-sectional design, mediation findings should be interpreted as exploratory and hypothesis-generating rather than causal.

To explore potential pathways underlying the association between the TyG index and hemorrhoidal disease, we performed exploratory mediation analyses with BMI and constipation as candidate mediators (**Figure 4**). When BMI was examined as the mediator, the indirect effect (ACME) via BMI was 0.142 (95% CI: 0.073–0.218), accounting for approximately 27.1% (95% CI: 16.4%–42.3%) of the total association, with a persistent direct effect (ADE) of 0.382 (95% CI: 0.241–0.525).

In contrast, constipation mediated only a modest proportion of the total effect, with an ACME of 0.049 (95% CI: 0.012–0.089), representing merely 9.4% (95% CI: 4.3%–18.5%) of the total effect (**Figure 4B**).

## 4 Discussion

The present cross-sectional study based on retrospectively collected clinical data identified a positive association between the TyG index and hemorrhoidal disease among adults undergoing anorectal evaluation. Participants in the highest TyG quartile had a substantially higher prevalence of hemorrhoidal disease than those in the lowest quartile, and the association remained significant after comprehensive adjustment for potential confounders. Each 1-unit increase in the TyG index was associated with greater odds of hemorrhoidal disease, with a significant graded trend across quartiles. Restricted cubic spline analysis indicated an approximately linear association, without strong evidence of nonlinearity. The results were consistent across subgroup and sensitivity analyses, and a higher TyG index was also associated with greater odds of advanced disease.

Previous epidemiological studies have shown that hemorrhoidal disease is associated with a complex combination of demographic, behavioral, gastrointestinal, and metabolic characteristics. Large health-screening studies have reported associations with age, sex, obesity, hypertension, smoking, and constipation, although the magnitude and consistency of individual associations have varied across populations (8, 9). A Mendelian randomization study further supported a potential role of adiposity in the development of hemorrhoids (10). These findings suggest that hemorrhoidal disease may not be explained exclusively by local mechanical factors, such as straining, prolonged defecation, or increased intra-abdominal pressure. Instead, systemic metabolic characteristics may contribute to individual susceptibility, either independently or through interactions with bowel habits and lifestyle.

The present results also extend limited evidence linking glucose and lipid abnormalities with hemorrhoidal disease. A recent investigation among patients with diabetes suggested that glycemic regulation and lipid profiles may be related to the occurrence and clinical characteristics of hemorrhoidal disease (11). However, diabetes represents a clinically diagnosed metabolic condition and may not capture the broader continuum of insulin resistance in individuals without overt hyperglycemia. The TyG index combines fasting glucose and triglyceride concentrations and has been validated as an accessible surrogate measure of insulin resistance (12, 13). Its association with hemorrhoidal disease in the present study therefore raises the possibility that subclinical glucose-lipid dysregulation may be relevant even before clinically apparent diabetes develops.

The observed findings are broadly consistent with studies relating the TyG index to vascular abnormalities. Higher TyG values have been associated with cardiovascular disease, arterial stiffness, coronary calcification, and endothelial dysfunction (14, 15). A clinical study using flow-mediated dilation found that an elevated TyG index was associated with impaired endothelial function, including among participants without diabetes (16). Insulin resistance itself has also been linked to impaired vasodilation, reduced nitric oxide bioavailability, oxidative stress, arterial stiffness, and subclinical vascular injury (17, 18). Although most of this evidence relates to the arterial circulation, it supports a broader interpretation of the TyG index as a marker of systemic vascular and metabolic dysfunction rather than merely a composite laboratory measure.

The mediation analyses in our study provide novel insights into the potential mechanistic pathways linking systemic metabolic dysfunction to hemorrhoidal disease. Notably, BMI mediated nearly 27% of the total TyG-hemorrhoid association. This finding is biologically coherent and clinically interpretable. A higher TyG index, as a surrogate of hyperinsulinemia and insulin resistance, directly promotes visceral adiposity and weight gain. The resultant increase in BMI elevates intra-abdominal pressure and central venous pressure, which may chronically impair venous return from the inferior vena cava and exacerbate venous congestion within the hemorrhoidal plexus. Furthermore, obesity-related adipokine dysregulation (e.g., leptin resistance, adiponectin deficiency) and low-grade systemic inflammation may compromise the structural integrity of the connective tissue supporting the anal cushions, facilitating both venous dilation and prolapse (19, 20).

Notably, a substantial proportion of the association remained unexplained after accounting for BMI and constipation, as indicated by the persistent ADE. This residual association may reflect biological pathways directly related to insulin resistance and lipid dysregulation captured by the TyG index. Hyperinsulinemia associated with insulin resistance may impair vascular endothelial homeostasis by reducing endothelial nitric oxide synthase (eNOS) activity and nitric oxide bioavailability, thereby promoting endothelial dysfunction, altered vascular tone, and impaired microcirculatory regulation within the hemorrhoidal plexus. Such metabolic disturbances may contribute to venous congestion and vascular remodeling, which are central features of hemorrhoidal disease development.

In addition, elevated triglyceride levels and chronic hyperglycemic exposure may induce lipotoxicity, oxidative stress, and extracellular matrix remodeling. Experimental studies have shown that metabolic stress can activate matrix metalloproteinases (particularly MMP-2 and MMP-9), which degrade collagen and elastic fiber components supporting vascular structures. In the anorectal region, disruption of the connective tissue framework of the anal cushions may facilitate their downward displacement and venous enlargement. Furthermore, TyG-related metabolic dysfunction is closely associated with chronic low-grade inflammation, including increased production of inflammatory mediators such as tumor necrosis factor-α and interleukin-6, which may further promote vascular permeability, inflammatory activation, and thrombotic susceptibility within hemorrhoidal vessels.

Interestingly, constipation explained only a modest proportion (approximately 9%) of the total effect, despite being a well-recognized clinical risk factor. This relatively small mediating proportion suggests that abnormal bowel habits are not the dominant bridge between glucose-lipid metabolic dysregulation and hemorrhoidal disease. More importantly, the substantial residual direct effect (ADE) that remained after accounting for both BMI and constipation implies that TyG may influence hemorrhoidal pathophysiology through additional pathways not captured in our analysis. These may include direct endothelial dysfunction with impaired nitric oxide bioavailability, chronic low-grade systemic inflammation, or microvascular remodeling—mechanisms previously documented in studies linking TyG to arterial stiffness and cardiovascular microvascular injury (14, 16). Therefore, rather than acting solely through mechanical pressure or straining, systemic metabolic dysfunction may exert a more fundamental vascular effect on the anorectal venous sinusoids.

The approximately linear pattern identified by restricted cubic spline analysis is also clinically relevant. The absence of a statistically supported threshold suggests that the association may extend across the measured TyG range rather than appearing only above a single cutoff. Accordingly, the present findings do not support defining a specific TyG value as a diagnostic threshold for hemorrhoidal disease. This is important because TyG distributions differ according to age, ethnicity, adiposity, diabetes prevalence, laboratory methods, and population characteristics. Previous investigations have similarly emphasized that the clinical interpretation of TyG should consider the study population and that universally accepted cutoff values have not been established (13, 14). The continuous association observed here may therefore be more informative than any single dichotomous classification.

Although the association appeared numerically stronger among older participants, those with higher BMI, and participants with constipation, none of the interaction tests reached statistical significance. These subgroup patterns should consequently be interpreted as exploratory rather than evidence of true effect modification. The direction of association was nevertheless broadly consistent across sex, age, BMI, diabetes, and constipation categories. This consistency, together with the sensitivity analyses excluding diabetes, medication use, and extreme TyG values, suggests that the main finding was not entirely attributable to overt diabetes or a small number of metabolically abnormal participants.

The association with hemorrhoidal severity provides additional, although preliminary, support for a possible relationship between metabolic dysfunction and the clinical expression of hemorrhoidal disease. Participants with higher TyG values had greater odds of grade III–IV disease after adjustment for available confounders. One possible explanation is that prolonged metabolic and vascular dysfunction may contribute not only to the presence of hemorrhoidal disease but also to vascular enlargement, tissue degeneration, and prolapse progression. However, severity was assessed only among affected participants, the number of advanced cases was limited, and the cross-sectional design could not determine whether elevated TyG preceded progression from early to advanced disease. This result should therefore be considered hypothesis-generating.

From a clinical perspective, fasting glucose and triglycerides are inexpensive and routinely measured, making the TyG index readily obtainable without additional testing. The findings suggest that patients with hemorrhoidal disease, particularly those with high TyG values, may benefit from assessment of their broader metabolic profile, including obesity, blood pressure, glycemic status, and dyslipidemia. However, TyG should not be interpreted as a diagnostic test for hemorrhoids or as a basis for selecting surgical treatment. Instead, it may provide complementary information regarding the systemic metabolic context in which hemorrhoidal disease occurs. Large prospective studies have shown that TyG is associated with future cardiovascular and metabolic outcomes (21), while recent work suggests that it may identify metabolic syndrome more effectively than some insulin-based measures in selected populations (22). Consequently, an elevated TyG index in a patient presenting with hemorrhoidal disease may justify attention to modifiable cardiometabolic factors, irrespective of whether those factors directly influence the anorectal condition.

The clinical implications should also be viewed in the context of shared risk factors. Adiposity, sedentary behavior, dietary patterns, bowel habits, hypertension, and glucose-lipid abnormalities may simultaneously influence both metabolic health and hemorrhoidal symptoms. Recent genetic epidemiological research has suggested possible relationships between adiposity, bowel characteristics, and hemorrhoids (10, 23). Conversely, a bidirectional Mendelian randomization analysis did not demonstrate a clear causal relationship between hemorrhoids and major cardiovascular diseases, suggesting that reported epidemiological associations may partly reflect shared determinants rather than a direct disease-to-disease pathway (24). This distinction reinforces the need to avoid interpreting hemorrhoidal disease as a cardiovascular manifestation or elevated TyG as a proven cause of hemorrhoids.

This study has several strengths. First, the TyG index was assessed both continuously and by quartiles, reducing dependence on a single exposure definition. Second, sequential multivariable models accounted for a broad range of demographic, metabolic, medication-related, lifestyle, and bowel-related variables. Third, restricted cubic spline analysis allowed the shape of the association to be examined without imposing a simple linear assumption. Fourth, subgroup and interaction analyses assessed the consistency of the findings across clinically relevant populations. Finally, multiple sensitivity analyses produced similar effect estimates, supporting the internal robustness of the primary results.

Several limitations should be acknowledged. First, selection bias and referral bias should be considered. Participants were recruited from a specialized proctology department and underwent anorectal evaluation because of clinical indications rather than population-based screening. Therefore, this cohort may have included individuals with greater symptom burden, healthcare-seeking behavior, or metabolic comorbidity than the general population. The observed hemorrhoidal disease detection rate (37.1%) should therefore be interpreted within this clinically evaluated population and should not be directly generalized to community-dwelling adults. In addition, because both metabolic abnormalities and anorectal symptoms may influence referral patterns, the magnitude of the observed association between the TyG index and hemorrhoidal disease may differ in unselected populations. Second, the retrospective cross-sectional design prevents determination of temporal sequence and causal relationships. Although a higher TyG index may contribute to hemorrhoidal disease through metabolic pathways, shared behavioral factors, reverse causation, or bidirectional relationships cannot be excluded. This limitation is particularly relevant for mediation analyses. Although BMI statistically accounted for approximately 27.1% and constipation approximately 9.4% of the observed association, these findings should be interpreted as exploratory and hypothesis-generating rather than evidence of confirmed causal mediation. BMI may represent an upstream determinant of insulin resistance rather than a downstream mediator, and longitudinal studies with repeated metabolic and anthropometric measurements are required. Third, the single-center design may limit external validity across different healthcare systems and populations. Although multiple demographic, metabolic, lifestyle, medication-related, and bowel-related factors were adjusted for, residual confounding from unmeasured or incompletely measured factors remains possible. These factors may include detailed dietary intake, duration and severity of constipation, defecation posture, occupational sitting or standing, pregnancy history, parity, family history, socioeconomic status, and medications affecting bowel function. Fourth, the TyG index was calculated from a single fasting measurement and may be influenced by short-term biological variation. Direct measures of insulin resistance, such as fasting insulin concentrations, homeostasis model assessment, or hyperinsulinemic-euglycemic clamp testing, were unavailable. Fifth, hemorrhoidal grading may involve interobserver variability, particularly in patients with mixed hemorrhoids, although diagnoses were independently reviewed by colorectal specialists. Sixth, the analysis of grade III–IV disease included fewer events than the primary outcome analysis and therefore yielded less precise estimates. Finally, the study did not directly assess endothelial function, inflammatory biomarkers, anorectal microcirculation, venous pressure, or tissue remodeling. Therefore, the proposed biological mechanisms involving insulin resistance, endothelial dysfunction, inflammation, and extracellular matrix remodeling remain plausible explanations rather than demonstrated pathways.

## 5 Conclusion

A higher TyG index was independently associated with greater odds of hemorrhoidal disease in this retrospective cross-sectional study. The association was approximately linear, remained generally consistent across subgroup and sensitivity analyses, and was also observed in relation to advanced disease severity. These findings suggest that glucose-lipid metabolic dysfunction may represent a relevant systemic correlate of hemorrhoidal disease. However, the results do not establish causality, temporal direction, or predictive utility. Prospective and mechanistic studies are needed to confirm the association and determine its biological and clinical significance.

## Data Availability

The original contributions presented in the study are included in the article/**Supplementary Material**. Further inquiries can be directed to the corresponding author.

## Supplementary material

Supplementary Table 1 Full list of candidate covariates considered *a priori* and final inclusion status

Supplementary Table 2 Missing data pattern of covariates included in multivariable analyses

Supplementary Table 3 Subgroup analyses of the association between TyG index and hemorrhoidal disease Note: Models were adjusted for age, sex, BMI, smoking, alcohol consumption, hypertension, diabetes mellitus, medication use, constipation, sedentary behavior, physical activity, and dietary fiber intake, excluding the stratification variable from the corresponding model.
