# Supplementary material for: Association between the triglyceride-glucose index and the presence and severity of hemorrhoidal disease: a cross-sectional study based on retrospectively collected clinical data
Source: Front Endocrinol (Lausanne). 2026 Aug 31;17:1935994. doi: 10.3389/fendo.2026.1935994 (PMC13572235; doi:10.3389/fendo.2026.1935994)
Supplement: Supplementary file 1 [file Table1.docx]

**Supplementary Table S1. Full list of candidate covariates considered a priori and final inclusion status**

| **Candidate variable** | **Included in Model 3/4?** | **Reason if excluded** |
| --- | --- | --- |
| Age | Yes | Prespecified demographic confounder |
| Sex | Yes | Prespecified demographic confounder |
| Body mass index | Yes | Prespecified metabolic confounder |
| Smoking status | Yes | Prespecified lifestyle confounder |
| Alcohol consumption | Yes | Prespecified lifestyle confounder |
| Hypertension | Yes | Prespecified metabolic confounder |
| Diabetes mellitus | Yes | Prespecified metabolic confounder |
| Glucose-lowering medication use | Yes | To account for treatment effects |
| Lipid-lowering medication use | Yes | To account for treatment effects |
| Constipation | Yes | Established clinical risk factor |
| Sedentary behaviour | Yes | Prespecified lifestyle confounder |
| Physical activity | Yes | Prespecified lifestyle confounder |
| Dietary fibre intake | Yes | Prespecified lifestyle confounder |
| Chronic diarrhoea | Yes | Bowel-habit confounder |
| Straining during defecation | Yes | Bowel-mechanical confounder |
| Prolonged toilet sitting | Yes | Bowel-mechanical confounder |
| Systolic blood pressure | No | Highly correlated with hypertension (VIF >5 in preliminary check) |
| Diastolic blood pressure | No | Highly correlated with hypertension |
| Cardiovascular disease | No | Low prevalence (n<10) in this cohort |
| Frequency of bowel movements | No | Incomplete/heterogeneous documentation in EHR |
| Family history of hemorrhoids | No | Not routinely recorded in EHR |

Supplementary Table S2. Missing data pattern of covariates included in multivariable analyses

| **Variable** | **Missing n** | **Missing (%)** |
| --- | --- | --- |
| Age | 0 | 0 |
| Sex | 0 | 0 |
| BMI | 11 | 2.1 |
| Smoking status | 8 | 1.5 |
| Alcohol consumption | 10 | 1.9 |
| Hypertension | 3 | 0.6 |
| Diabetes mellitus | 2 | 0.4 |
| Lipid-lowering medication use | 6 | 1.1 |
| Glucose-lowering medication use | 4 | 0.8 |
| Physical activity | 18 | 3.4 |
| Sedentary behavior | 15 | 2.8 |
| Dietary fiber intake | 21 | 4 |
| Constipation | 12 | 2.3 |
| Chronic diarrhea | 7 | 1.3 |
| Straining during defecation | 16 | 3 |
| Prolonged toilet sitting | 14 | 2.7 |

No prespecified covariate had missingness exceeding 20%. Therefore, all eligible missing covariate values were handled using MICE in the primary analysis.

**Supplementary Table S3. Subgroup analyses of the association between TyG index and hemorrhoidal disease**

| **Subgroup** | **Participants, n** | **Adjusted OR per 1-unit increase (95% CI)** | **P value** | **P for interaction** |
| --- | --- | --- | --- | --- |
| Sex |  |  |  | 0.724 |
| Male | 304 | 1.72 (1.14–2.60) | 0.01 |  |
| Female | 224 | 1.61 (1.02–2.55) | 0.041 |  |
| Age |  |  |  | 0.438 |
| <45 years | 239 | 1.49 (0.91–2.44) | 0.112 |  |
| ≥45 years | 289 | 1.83 (1.23–2.72) | 0.003 |  |
| BMI |  |  |  | 0.286 |
| <24 kg/m² | 241 | 1.46 (0.91–2.34) | 0.117 |  |
| ≥24 kg/m² | 287 | 1.86 (1.24–2.79) | 0.003 |  |
| Diabetes mellitus |  |  |  | 0.643 |
| No | 471 | 1.65 (1.19–2.28) | 0.003 |  |
| Yes | 57 | 1.79 (0.77–4.16) | 0.177 |  |
| Constipation |  |  |  | 0.361 |
| No | 366 | 1.51 (1.02–2.24) | 0.039 |  |
| Yes | 162 | 1.92 (1.19–3.10) | 0.008 |  |

Note: Models were adjusted for age, sex, BMI, smoking, alcohol consumption, hypertension, diabetes mellitus, medication use, constipation, sedentary behavior, physical activity, and dietary fiber intake, excluding the stratification variable from the corresponding model.
